# Data on the sequence-derived properties of gastric cancer – binding peptides

**DOI:** 10.1016/j.dib.2020.105351

**Published:** 2020-02-29

**Authors:** Jose Isagani B. Janairo, Marianne Linley L. Sy-Janairo

**Affiliations:** a Biology Department, De La Salle University, 2401 Taft Avenue, Manila, Philippines; b Institute of Digestive and Liver Diseases, St. Luke's Medical Center – Global City, Taguig, Philippines

**Keywords:** Medical bioinformatics, Therapeutic peptides, Drug design and discovery, Peptide descriptors

## Abstract

The article presents a dataset containing nine classes of calculated sequence-derived descriptors for 78 peptide sequences, 21 of which demonstrate the ability to bind with gastric cancer cells. The datasaet was used in the paper “A screening algorithm for gastric cancer binding peptides” [1] for the creation of a classification model that can predict the ability of a given peptide sequence to bind with gastric cancer cells. The 78 peptide sequences were extracted from a systematic literature search, and the various peptide descriptors were calculated using the R package “Peptides”. The nine calculated sequence-derived descriptor classes are the Blosum indices, Cruciani properties, FASGAI vectors, Kidera factors, ProtFP, ST-scales, T-scales, VHSE scales, and Z-scales. The resulting dataset, which is composed of over 4000 data points, offers a rich resource for further protochemometric analyses of the curated peptide sequences relevant to cancer diagnostics and therapeutics.

**Table:** Specifications Table

Subject	Biochemistry, genetics, and molecular biology
Specific subject area	Peptide bioinformatics
Type of data	Table
How data were acquired	Systematic literature search followed by *in silico* peptide sequence-dependent calculations using the “Peptides” R package.
Data format	Raw and processed
Parameters for data collection	The nine calculated sequence-derived descriptor classes are the Blosum indices, Cruciani properties, FASGAI vectors, Kidera factors, ProtFP, ST-scales, T-scales, VHSE scales, and Z-scales. These descriptors were then used to create classifiers for gastric cancer-binding peptides using logistic regression, classification and regression trees, support vector machine, and random forest.
Description of data collection	A thorough literature search in major publication databases was conducted to search for studies that report binding and non-binding peptides for gastric cancer cell. The different sequence-dependent properties of extracted peptide sequences were then calculated, and were used for the creation of classification models using different algorithms.
Data source location	De La Salle University, Manila, Philippines
Data accessibility	With the article
Related research article	Jose Isagani B. Janairo, Marianne Linley L. Sy-Janairo, A screening algorithm for gastric cancer – binding peptides, International Journal of Peptide Research and Therapeutics, https://doi.org/10.1007/s10989-019-09874-8

**Table:** 

**Value of the Data** • The data composed of more than 4000 data points, presents systematically curated peptide sequences relevant to gastric cancer diagnostics together with a comprehensive array of sequence-derived peptide descriptors. • The data together with the code save interested researchers the time and effort for searching peptide sequences associated with gastric cancer cell- binding and their sequence-based properties, which can be further used for machine learning applications, experimental validations, among others. • Researchers in medicinal chemistry, biochemistry, medicine, computational biology, and allied fields may find the data useful for further discovery of gastric cancer – binding peptides, rational design of such peptide class, pattern recognition, QSAR modelling, among others.

## 1 Data description

The dataset is composed of 21 gastric cancer – binding peptide (GCBP) sequences, and 57 non-GCBP [[2], [3], [4], [5], [6], [7], [8]]. The dataset was used in [1] for the creation of a classification algorithm that can categorize peptide sequences into GCBP and non-GCBP. Nine sequence-dependent descriptor classes were calculated for each peptide, and the consolidated dataset is available in the supporting information. In the table, the columns with the heading Blosum 1–10 each represents the calculated ten Blosum indices, CP 1–3 for the three Cruciani properties, F 1–6 for the six FASGAI vectors, KF 1–10 for the ten Kidera factors, ProtFP 1–8 for the eight ProtFP descriptors, ST 1–8 for the eight ST-scales, T 1–5 for the five T-scales, VHSE 1–8 for the eight VHSE scales, and Z 1–5 for the five Z-scales. The nine peptide descriptor classes can be categorized depending on what they are describing. The Blosum indices are similarity descriptors, the T-scales and ST-scales are topological descriptors; the FASGAI vectors, ProtFP, VHSE scales, and Z-scales are physico-chemical descriptors; and the Cruciani properties and Kidera factors are combination of descriptor classes [9]. The average training accuracy, as well as the accuracy per fold using the different descriptors and algorithms are presented in Table 1, Table 2, Table 3, Table 4, Table 5, Table 6, Table 7, Table 8, Table 9.

**Table 1:** Training accuracy (%) of different classification algorithms using Blosum indices as the descriptor.

Training Accuracy	Logistic Regression	Classification and Regression Trees	k-Nearest Neighbor	Support VectorMachine	Random Forest
Fold 1	100	75	75	67	67
Fold 2	20	80	75	67	75
Fold 3	100	80	25	80	83
Fold 4	100	50	83	75	80
Fold 5	80	60	67	75	50
Fold 6	60	60	80	75	80
Fold 7	60	80	75	75	80
Fold 8	80	60	67	75	75
Fold 9	50	50	75	80	75
Fold 10	60	67	80	67	100
Average	71	66	70	74	77

**Table 2:** Training accuracy (%) of different classification algorithms using Cruciani properties as the descriptor.

Training Accuracy	Logistic Regression	Classification and Regression Trees	k-Nearest Neighbor	Support Vector Machine	Random Forest
Fold 1	80	50	100	67	75
Fold 2	40	40	100	67	83
Fold 3	100	40	50	80	50
Fold 4	75	75	60	75	100
Fold 5	80	80	50	75	75
Fold 6	80	60	100	75	80
Fold 7	40	80	75	75	80
Fold 8	80	80	80	75	20
Fold 9	50	75	75	80	75
Fold 10	60	67	83	67	75
Average	69	63	77	74	71

**Table 3:** Training accuracy (%) of different classification algorithms using FASGAI vectors as the descriptor.

Training Accuracy	Logistic Regression	Classification and Regression Trees	k-Nearest Neighbor	Support Vector Machine	Random Forest
Fold 1	60	75	75	67	67
Fold 2	60	0	50	67	75
Fold 3	80	80	50	80	60
Fold 4	75	75	67	75	67
Fold 5	80	40	67	75	40
Fold 6	60	40	80	75	50
Fold 7	40	75	75	75	60
Fold 8	60	60	67	75	80
Fold 9	50	80	75	80	75
Fold 10	40	50	80	67	75
Average	61	58	69	74	65

**Table 4:** Training accuracy (%) of different classification algorithms using Kidera factors as the descriptor.

Training Accuracy	Logistic Regression	Classification and Regression Trees	k-Nearest Neighbor	Support Vector Machine	Random Forest
Fold 1	80	40	75	67	67
Fold 2	60	75	50	67	75
Fold 3	60	75	50	80	80
Fold 4	75	100	67	75	50
Fold 5	80	80	67	75	60
Fold 6	40	40	80	75	100
Fold 7	60	80	75	75	80
Fold 8	40	100	67	75	60
Fold 9	50	67	75	80	75
Fold 10	60	20	80	67	75
Average	61	68	69	74	72

**Table 5:** Training accuracy (%) of different classification algorithms using protFP as the descriptor.

Training Accuracy	Logistic Regression	Classification and Regression Trees	k-Nearest Neighbor	Support Vector Machine	Random Forest
Fold 1	80	50	75	67	50
Fold 2	60	40	75	67	75
Fold 3	80	40	83	80	60
Fold 4	75	75	60	75	67
Fold 5	80	80	75	75	60
Fold 6	40	60	67	75	75
Fold 7	60	80	75	75	100
Fold 8	60	20	80	75	80
Fold 9	50	75	75	80	75
Fold 10	60	67	67	67	75
Average	65	59	73	74	72

**Table 6:** Training accuracy (%) of different classification algorithms using stScales as the descriptor.

Training Accuracy	Logistic Regression	Classification and Regression Trees	k-Nearest Neighbor	Support Vector Machine	Random Forest
Fold 1	80	40	75	67	100
Fold 2	60	75	75	67	83
Fold 3	100	75	25	80	75
Fold 4	75	100	67	75	40
Fold 5	80	60	50	75	83
Fold 6	20	40	60	75	80
Fold 7	60	60	75	75	75
Fold 8	80	400	50	75	60
Fold 9	50	33	75	80	60
Fold 10	60	80	80	67	100
Average	67	66	63	74	76

**Table 7:** Training accuracy (%) of different classification algorithms using tScales as the descriptor.

Training Accuracy	Logistic Regression	Classification and Regression Trees	k-Nearest Neighbor	Support Vector Machine	Random Forest
Fold 1	100	60	100	67	83
Fold 2	60	50	75	67	75
Fold 3	100	100	50	80	60
Fold 4	100	60	80	75	50
Fold 5	50	40	50	75	80
Fold 6	60	50	67	75	75
Fold 7	60	75	75	75	60
Fold 8	80	50	80	75	60
Fold 9	100	100	100	80	100
Fold 10	60	80	33	67	75
Average	800	67	71	74	72

**Table 8:** Training accuracy (%) of different classification algorithms using VHSEScales as the descriptor.

Training Accuracy	Logistic Regression	Classification and Regression Trees	k-Nearest Neighbor	Support Vector Machine	Random Forest
Fold 1	40	80	75	67	67
Fold 2	80	50	75	67	75
Fold 3	80	25	75	80	67
Fold 4	75	60	67	75	80
Fold 5	80	60	67	75	75
Fold 6	100	80	80	75	80
Fold 7	60	80	75	75	80
Fold 8	60	75	67	75	75
Fold 9	50	50	75	80	50
Fold 10	60	80	80	67	60
Average	69	64	74	74	71

**Table 9:** Training accuracy (%) of different classification algorithms using zScales as the descriptor.

Training Accuracy	Logistic Regression	Classification and Regression Trees	k-Nearest Neighbor	Support Vector Machine	Random Forest
Fold 1	40	50	75	67	50
Fold 2	80	80	100	67	25
Fold 3	80	80	83	80	80
Fold 4	100	75	80	75	67
Fold 5	80	80	75	75	60
Fold 6	60	60	67	75	100
Fold 7	60	60	75	75	80
Fold 8	60	60	40	75	80
Fold 9	75	75	75	80	75
Fold 10	60	67	67	67	50
Average	70	69	74	74	67

## 2 Experimental design, materials, and methods

Peptide sequences that can bind or cannot bind with gastric cancer cells were systematically searched in major databases such as Scopus, Google Scholar, and Pubmed. A given peptide sequence was identified as GCBP if the paper reports a statistically higher binding affinity compared with a control. If the reported peptide sequence did not exhibit statistical difference with a control, it was classified as non-GCBP. The identified 78 peptide sequences, 21 of which are GCBP and 57 non-GCBP, were then used to calculate nine sequence-dependent peptide descriptor classes using the R package “Peptides” version 2.4 [10], executed in R version 3.5.2 using a Windows 64 bit desktop [11]. The nine calculated sequence-derived descriptor classes are the Blosum indices, Cruciani properties, FASGAI vectors, Kidera factors, ProtFP, ST-scales, T-scales, VHSE scales, and Z-scales. These descriptors were then used to train classification models using logistic regression, classification and regression trees, k-nearest neighbor, support vector machine, and random forest in R. Sixty percent of the dataset was dedicated for training the classification model, and the remaining forty percent for testing the model performance. The codes or R scripts used in calculating the peptide descriptors for the 78 peptide sequences, and the code for training the classifiers are available in the supporting information. Each R script is named after the descriptor class that is being calculated. Should the accompanying R scripts be used, the working directory should be changed accordingly.

## Conflict of Interest

The authors declare that they have no known competing financial interests or personal relationships that could have appeared to influence the work reported in this paper.
